# Development of Microsatellite Markers in *Pungtungia herzi* Using Next-Generation Sequencing and Cross-Species Amplification in the Genus *Pseudopungtungia*

**DOI:** 10.3390/ijms141019923

**Published:** 2013-10-01

**Authors:** Young-Eun Yun, Jeong-Nam Yu, Sang Ki Kim, Ui Wook Hwang, Myounghai Kwak

**Affiliations:** 1 National Institute of Biological Resources, Incheon 404-708, Korea; E-Mails: woo0711@korea.kr (Y.-E.Y.); susia000@korea.kr (J.-N.Y.); 2 School of Life Science, Kyungpook National University, Daegu 702-701, Korea; E-Mails: ivoice@hanmail.net (S.K.K.); uwhwang@knu.ac.kr (U.W.H.); 3 Department of Biology, Teachers College & Institute for Phylogenomics and Evolution, Kyungpook National University, Daegu 702-701, Korea

**Keywords:** microsatellite, *Pungtungia herzi*, *Pseudopungtungia*, cross-species amplification, next-generation sequencing

## Abstract

Nuclear microsatellite markers for *Pungtungia herzi* were developed using a combination of next-generation sequencing and Sanger sequencing. One hundred primer sets in the flanking region of dinucleotide and trinucleotide repeat motifs were designed and tested for efficiency in polymerase chain reaction amplification. Of these primer sets, 16 new markers (16%) were successfully amplified with unambiguous polymorphic alleles in 16 individuals of *Pungtungia herzi*. Cross-species amplification with these markers was then examined in two related species, *Pseudopungtungia nigra* and *Pseudopungtungia tenuicorpa*. Fifteen and 11 primer pairs resulted in successful amplification in *Pseudopungtungia nigra* and *Pseudopungtungia tenuicorpa*, respectively, with various polymorphisms, ranging from one allele (monomorphic) to 11 alleles per marker. These results indicated that developing microsatellite markers for cross-amplification from a species that is abundant and phylogenetically close to the species of interest is a good alternative when tissue samples of an endangered species are insufficient to develop microsatellites.

## 1. Introduction

The Korean endemic genus *Pseudopungtungia* includes two endangered species, the black shinner (*Pseudopungtungia nigra*) and the slender shinner (*Pseudopungtungia tenuicorpa*), that have suffered drastic population decreases [1,2]. *Pseudopungtungia* is restricted to the Geum, Mankyung, Ungcheon, and Han Rivers in South Korea [1,2]. The population declines are attributed to habitat destruction, including dam construction, and to a population decrease of the host species, *Coreoperca herzi* (Perciformes: Centropomidae) for brood parasites [3–5].

A good understanding of the genetics of endangered species can assist in developing strategies for their conservation [6]; however, to date, no population genetic studies of these species have been performed and no high-resolution molecular markers have been developed for them. Insufficient genomic DNA and the lack of an appropriate number of individuals to screen polymorphic markers hinder further research progress. To overcome this difficulty, we used transferable microsatellite markers from a phylogenetically close species. Recent studies have demonstrated the value of the cross-species microsatellite amplification approach in population studies of species for which microsatellites have not yet been developed [7,8]. The limitations of cross-species microsatellite amplification are reduced levels of diversity in the target species [9], increased frequency of null alleles and homoplasy [10–12], and limited applicability to very closely related species belonging to the same genus or to recently separated genera [13,14].

The striped shiner (*Pungtungia herzi*) is widely distributed in most rivers and lakes in South Korea, northern China, and southern Japan [1,15]. It reaches lengths of 10–15 cm and feeds on attached algae and aquatic insect larvae. *Pungtungia herzi* uses two reproductive strategies, nonparasitic crevice spawning and brood parasitism with multiple host species, including *Coreoperca herzi*, *C. kawamebari*, *Pseudobagrus nudicepts*, and *Odontobutis obscura* (Perciformes: Odontobutidae) [16–19]. *Pungtungia herzi* and *Pseudopungtungia nigra* appear to be closely related phylogenetically, due to their morphological similarities and rather distinct zoogeographic distributions [20]. In fact, a probable natural hybrid of *Pungtungia herzi* and *Pseudopungtungia nigra* was reported in the Ungcheon River [15].

Next-generation sequencing (NGS) methods are revolutionizing molecular ecology by simplifying the development of molecular genetic markers, including microsatellites [21]. In this study, we developed a set of microsatellite markers from *Pungtungia herzi* using a NGS protocol [22]. We then examined the polymorphic markers from *Pungtungia herzi* in the two related endemic species, *Pseudopungtungia nigra* and *Pseudopungtungia tenuicorpa*.

## 2. Results and Discussion

### 2.1. Results of DNA Sequencing with A Roche 454 GS-FLX Titanium Run

We obtained 243,749 total reads (91.87 Mb) for *Pungtungia herzi*. The total number of contigs was 9708 and the number of singletons was 146,093. A total of 1981 regions containing perfect dinucleotide/trinucleotide repeats were identified. The most frequent dinucleotide type in the *Pungtungia herzi* genome was CA repeats (51.66%), followed by AT (25.10%), CT (21.77%), and GC (1.47%). These results are consistent with previous findings that the CA repeat is the most frequent in 353 species of vertebrata genomes and is generally 2.3-fold more frequent than the second most common microsatellite, AT [23]. Of the trinucleotide repeats, the AAT/ATA/TAA motif was the most frequent (59.87%), and the CGG/GCG/GGC repeat motif was the least common. Among the isolated dinucleotide microsatellites, the highest number of repeat-unit iterations was 27 (54 bp); among the trinucleotide microsatellites, the highest number of repeat-unit iterations was 19 (57 bp).

### 2.2. Microsatellite Marker Development

We chose 100 microsatellites with the largest number of repeat motifs to test amplification efficiency and assess polymorphism. Of these, 16 new markers (16%) produced strongly amplified polymerase chain reaction (PCR) products in all specimens with unambiguous alleles in *Pungtungia herzi*. The nucleotide sequences of the 16 new microsatellite markers were deposited in the GenBank database under accession numbers JQ889798–JX915813 (Table 1).

Among three populations of *Pungtungia herzi*, these 16 markers were polymorphic (Table 2). The Gyeongho River population yielded 5–15 alleles, and the *H*_O_ and *H*_E_ ranged from 0.469 to 0.969 and from 0.580 to 0.913, respectively. The Imjin River population produced 3–15 alleles, and the Geum River population yielded 1–10 alleles.

Hardy–Weinberg equilibrium (HWE) was tested in the Gyeongho River population. In this population, PH_CA36, PH_CA38, PH_ATC01 (*p* < 0.01), and PH_CT03, PH_CA37, PH_ACT01 (*p* < 0.05) departed from HWE (Table 2). Of the markers, PH_CA38 and PH_ATC01 departed significantly from HWE and showed homozygote excess by analysis with MICRO-CHECKER. In general, the presence of null alleles or large allele dropout can explain observed deviation from HWE [9]. Finally, 10 new markers satisfied all the criteria (*i.e*., moderate to high polymorphism, no evidence of null alleles) and are recommended for use in future population genetics studies of *Pungtungia herzi* (Table 3).

### 2.3. Cross-Species Amplification

Cross-species amplification was further examined in *Pseudopungtungia nigra* and *Pseudopungtungia tenuicorpa*. Of the 16 new makers, the 10 markers that were strongly amplified in both *Pseudopungtungia nigra* and *Pseudopungtungia tenuicorpa*, 5 markers were amplified only in *Pseudopungtungia nigra*, while 1 marker (PH_ATC01) was amplified only in *Pseudopungtungia tenuicorpa*. The number of alleles for the 15 markers amplified in *Pseudopungtungia nigra* ranged from 1 to 11. The number of alleles on the 11 markers from *Pseudopungtungia tenuicorpa* ranged from 1 to 6 (Table 3). PH_CA41 was monomorphic in *Pseudopungtungia nigra*, whereas PH_CA52 and PH_TCA01 were monomorphic in *Pseudopungtungia tenuicorpa*. Consequently, 14 markers for *Pseudopungtungia nigra* and 9 markers for *Pseudopungtungia tenuicorpa* were considered to be good choices for future research in this area.

## 3. Experimental Section

### 3.1. Roche 454 GS-FLX Titanium Sequencing

Genomic DNA was extracted from muscle tissue using a DNeasy Blood and Tissue kit (QIAGEN, Hilden, Germany) following the manufacturer’s protocol and stored at −20 °C. The quality of genomic DNA was checked on 1% agarose gels with a spectrophotometer (NanoDrop Technologies, Wilmington, DE, USA). The NGS libraries were generated with aliquots of approximately 10 μg of genomic DNA from a single *Pungtungia herzi* individual and then sequenced using a 1/4 plate on the Roche 454 GS-FLX titanium platform at the National Instrumentation Center for Environmental Management of Seoul National University.

### 3.2. Microsatellite Marker Development

The sequence reads from the 454 GS-FLX were assembled using Newbler 2.6 (Roche Diagnostics, 454 Life Science, Mannheim, Germany) with a 96% minimum overlap identity. The dinucleotide and trinucleotide repeats of more than four iterations were searched using the perl program “ssr_finder.pl” [22,24]. These repeats were sorted and a pair of primers flanking each repeat was designed using Primer3 [25]. The optimal primer size was set to a range of 18–26 bases and the optimal melting temperature was set to 55–59 °C. The optimal product size was set to 130–270 bp and the remaining parameters were left at the default settings. For each primer pair, a 5′-M13 tail (5′-TGTAAAACGACGGCCAGT-3′) was added to the forward primer to allow fluorescent labeling during the amplification reactions [26].

### 3.3. PCR and Genotyping

The PCR mix contained 10 ng of genomic DNA, 8 pmol each of the reverse primer and fluorescent labeled (6-FAM, NED, PET, and VIC) M13 primer, and 4 pmol of the forward primer, Premix Taq (TaKaRa Ex Taq^®^ version 2.0; TaKaRa Bio, Otsu, Japan) in a final reaction volume of 20 μL. The PCR amplification protocol was 94 °C for 5 min, followed by 30 cycles of 94 °C for 30 s, 56 °C for 45 s, and 72 °C for 45 s, then 12 cycles of 94 °C for 30 s, 53 °C for 45 s, and 72 °C for 45 s, and a final extension at 72 °C for 10 min. Subsequently, the PCR product was added to Hi-Di™ formamide with 500 LIZ^®^ size standard (Applied Biosystems, Foster City, CA, USA) and run on an ABI 3730xl automatic sequencer (Applied Biosystems, Foster City, CA, USA). The microsatellite profiles were examined using GeneMarker ver. 1.85 (Softgenetics, State College, PA, USA). To verify the flanking sequences of the microsatellite repeats and to confirm whether target fragments of interest were amplified, PCR was performed with 8 pmol of forward and reverse primers without fluorescent-labeled M13 primer. The amplified products were sequenced using an ABI 3730XL and the targeted sequences were checked for correct amplification.

### 3.4. Statistical Analysis

We estimated the proportion of polymorphic markers and the average number of alleles per marker and calculated *H*_O_ and *H*_E_ using Arlequin ver. 3.11 [27]. Hardy-Weinberg equilibrium (HWE) in the Gyeongho River population of *Pungtungia herzi* was analyzed and the program MICRO-CHECKER ver. 2.2.3 [28] was used to check for null alleles and scoring errors due to stuttering or large allele dropout.

## 4. Conclusions

We developed 16 new microsatellite markers in *Pungtungia herzi*. Of these, 15 and 11 markers were applied successfully in the endangered species *Pseudopungtungia nigra* and *Pseudopungtungia tenuicorpa*, respectively. This achievement demonstrates that the microsatellite markers developed using the NGS method from a related species can be applied effectively to an endangered species. Developing these microsatellite primers will assist work to determine genetic relationships among the species, as well as improving our understanding of the population genetic structure within each species.

## Figures and Tables

**Table 1:** The set of 16 microsatellite markers developed in *Pungtungia herzi*.

Marker name	Repeat motifs	Primer sequence (5′-3′)	Annealing (°C)	Expected Size (bp)	GenBank accession No.
PH_CT03	GA_(14)_	F: M13-CAGAGCGTGAAAATGTCACTTA R: TAGTATCCCTCAAAGGTCAACG	56	153	JQ889798
PH_CT04	CT_(13)_	F: M13-GTATTCCAGGGAAAGTAGGAGG R: ACAGATTCACTCATAAGGGAGG	56	174	JQ889799
PH_CA12	TG_(17)_	F: M13-CAAGATGTGTGCTGGTTCTTTA R: ATCAGCAGTGCTAAGCTGAAGT	56	169	JQ889802
PH_CA28	CA_(16)_	F: M13-GACAGAGCTTGGTGACAGTTTT R: AAACATTACGTCTATGGCGAGT	56	172	JQ889804
PH_CA34	TG_(13)_	F: M13-CACCCTCTTCCCTTCAAGATA R: AGAAAGATTACGTCTGCCTCAG	56	166	JQ889805
PH_CA36	CA_(13)_	F: M13-CACCACTCCATGTACGGTATAG R: GTTTATGTCAGAGCCCAAATTC	56	171	JQ889806
PH_CA37	CA_(13)_	F: M13-GCGTTATCCATGAGAAGAACAT R: CTTACAGAACAGTGGGATTTGG	56	178	JQ889807
PH_CA38	CA_(13)_	F: M13-ACACTTTAAAAACCGACAGACG R: AAAATACCAATTAAAGGAGACGC	56	169	JQ889808
PH_CA41	CA_(12)_	F: M13-AGGTCCCAATTTGAGTTAAAAA R: ATCACTGTCTGATAAGTGTGCC	56	176	JX915806
PH_CA43	TG_(12)_	F: M13-CCTCACTGGCAATGAAGTCT R: TTTCTTTATTTCTCTCCCCCTC	56	146	JX915807
PH_CA46	TG_(12)_	F: M13-GGTTTGATTCAGTGTTGCACTA R: GTTATTTGTGGAGTGTTGGGAT	56	177	JX915808
PH_CA52	CA_(12)_	F: M13-AGAGATTTTCCCACCTACTGCT R: AGTGTGTACACCTCCTTGTGTG	56	148	JX915809
PH_CA54	CA_(12)_	F: M13-TATTTTTAACTGGCGCTAGGAC R: CATTTGACTGACTTGCAACAAT	56	145	JX915810
PH_TCA01	TCA_(13)_	F: M13-TAAGGCCCTACACCTGGTATTA R: TGTTTACACCTGAGACAAGTGG	56	174	JX915811
PH_ACT01	ACT_(15)_	F: M13-GTACAAGTGAGCTAAAGTGACAA R: AAACCAGATAGGGTCAAACATC	56	183	JX915812
PH_ATC01	ATC_(19)_	F: M13-CAGTGTCCAGTACTGTTCGTGT R: ATGGGGAGCTAAATTTGTAAT	56	193	JX915813

The “expected size” included 18 bp of the M13 tail.

**Table 2:** Polymorphisms of 16 microsatellite markers developed in *Pungtungia herzi*.

Marker name	Gyeongho river (*n* = 32)				Imjin river (*n* = 8)				Geum river (*n* = 8)			

	Size range	*N*_a_	*H*_O_	*H*_E_	Size range	*N*_a_	*H*_O_	*H*_E_	Size range	*N*_a_	*H*_O_	*H*_E_
PH_CT03	143–179	10	0.938	0.858 ^*^	145–149	3	0.750	0.692	145–147	2	0.375	0.325
PH_CT04	163–201	14	0.875	0.872	163–193	11	0.875	0.950	163–167	3	0.875	0.575
PH_CA12	167–187	12	0.875	0.852	160–187	10	0.750	0.933	158–230	10	0.625	0.892
PH_CA28	164–189	10	0.969	0.857	162–226	8	0.875	0.933	177–214	10	1.000	0.942
PH_CA34	161–225	12	0.844	0.813	163–184	9	0.500	0.908	163–190	8	0.750	0.900
PH_CA36	156–170	10	0.563	0.721 ^**^	152–176	7	0.625	0.850	162–174	6	0.625	0.850
PH_CA37	169–187	8	0.656	0.747 ^*^	169–200	7	0.750	0.825	179–196	7	0.875	0.850
PH_CA38	161–171	5	0.594	0.799 ^**^	156–173	9	0.750	0.892	156–160	3	0.500	0.492
PH_CA41	167–187	10	0.688	0.691	184–209	9	1.000	0.925	184–206	10	1.000	0.933
PH_CA43	135–153	8	0.750	0.751	132–143	5	0.375	0.850	136–145	4	0.750	0.725
PH_CA46	187–232	15	0.938	0.913	180–259	15	1.000	0.992	197–248	10	0.750	0.925
PH_CA52	139–177	13	0.844	0.832	151–178	11	1.000	0.950	164–170	3	0.750	0.700
PH_CA54	139–147	5	0.469	0.580	143–157	8	1.000	0.900	139–149	6	0.875	0.833
PH_TCA01	149–193	9	0.781	0.776	155–190	6	0.875	0.842	166–172	2	0.250	0.533
PH_ACT01	161–218	13	0.781	0.871 ^*^	161–206	7	0.875	0.867	164–173	4	0.250	0.517
PH_ATC01	161–188	11	0.594	0.862 ^**^	163–181	4	0.250	0.733	157–181	5	0.625	0.667

*n*, number of samples; *N*_a_, number of alleles; *H*_O_, observed heterozygosity; *H*_E_, expected heterozygosity. ^*^p < 0.05, ^**^p < 0.01: Significant departure from Hardy–Weinberg equilibrium in *Pungtungia herzi* in the Gyeongho River population. HW analysis of the Imjin and Geum Rivers were not performed because the sample size was too small. The size ranges include 18 bp of the M13 tail.

**Table 3:** Cross-species amplification of 16 microsatellite markers in *Pseudopungtungia nigra* (*n* = 6) and *Pseudopungtungia tenuicorpa* (*n* = 3).

Marker name	*Pseudopungtungia nigra*(Size range, *N*_a_)	*Pseudopungtungia tenuicorpa* (Size range, *N*_a_)
PH_CT03	+(153–175, 7)	+(150–160, 4)
PH_CT04	+(165–171, 4)	+(173–175, 2)
PH_CA12	+(162–212, 8)	−
PH_CA28	+(198–252, 10)	−
PH_CA34	+(157–174, 7)	+(150–176, 5)
PH_CA36	+(150–156, 4)	−
PH_CA37	+(166–190, 3)	+(173–175, 2)
PH_CA38	+(160–168, 4)	+(154–168, 5)
PH_CA41	+(169, 1)	+(172–192, 6)
PH_CA43	+(136–140, 2)	+(132, 1)
PH_CA46	+(194–258, 11)	−
PH_CA52	+(147–163, 5)	+(128, 1)
PH_CA54	+(147–161, 6)	+(155–191, 5)
PH_TCA01	+(155–158, 2)	+(163, 1)
PH_ACT01	+(169–181, 4)	−
PH_ATC01	−	+(191, 1)

*N*_a_, number of alleles; +, successful amplification; −, no amplification.
